# Acetylshikonin induces necroptosis via the RIPK1/RIPK3-dependent pathway in lung cancer

**DOI:** 10.18632/aging.205316

**Published:** 2023-12-19

**Authors:** Shih-Sen Lin, Tsung-Ming Chang, Augusta I-Chin Wei, Chiang-Wen Lee, Zih-Chan Lin, Yao-Chang Chiang, Miao-Ching Chi, Ju-Fang Liu

**Affiliations:** 1 Division of Chest Medicine, Department of Internal Medicine, Shin Kong Wu Ho-Su Memorial Hospital, Taipei 11101, Taiwan; 2 Translational Medicine Center, Shin-Kong Wu Ho-Su Memorial Hospital, Taipei 11101, Taiwan; 3 Department of Orthopaedic Surgery, Chang Gung Memorial Hospital, Puzi City 613016, Taiwan; 4 Department of Nursing, Division of Basic Medical Sciences, Chronic Diseases and Health Promotion Research Center, Chang Gung University of Science and Technology, Puzi City 613016, Taiwan; 5 Department of Safety Health and Environmental Engineering, Ming Chi University of Technology, New Taipei City 243303, Taiwan; 6 Division of Pulmonary and Critical Care Medicine, Chang Gung Memorial Hospital, Chiayi 613016, Taiwan; 7 Department of Respiratory Care, Chang Gung University of Science and Technology, Chiayi 613016, Taiwan; 8 School of Oral Hygiene, College of Oral Medicine, Taipei Medical University, Taipei 110301, Taiwan; 9 Department of Medical Research, China Medical University Hospital, China Medical University, Taichung 404328, Taiwan

**Keywords:** human lung cancer, acetylshikonin, ROS, necroptosis

## Abstract

Despite advances in therapeutic strategies, lung cancer remains the leading cause of cancer-related death worldwide. Acetylshikonin is a derivative of the traditional Chinese medicine Zicao and presents a variety of anticancer properties. However, the effects of acetylshikonin on lung cancer have not been fully understood yet. This study explored the mechanisms underlying acetylshikonin-induced cell death in non-small cell lung cancer (NSCLC). Treating NSCLC cells with acetylshikonin significantly reduced cell viability, as evidenced by chromatin condensation and the appearance of cell debris. Acetylshikonin has also been shown to increase cell membrane permeability and induce cell swelling, leading to an increase in the population of necrotic cells. When investigating the mechanisms underlying acetylshikonin-induced cell death, we discovered that acetylshikonin promoted oxidative stress, decreased mitochondrial membrane potential, and promoted G2/M phase arrest in lung cancer cells. The damage to NSCLC cells induced by acetylshikonin resembled results involving alterations in the cell membrane and mitochondrial morphology. Our analysis of oxidative stress revealed that acetylshikonin induced lipid oxidation and down-regulated the expression of glutathione peroxidase 4 (GPX4), which has been associated with necroptosis. We also determined that acetylshikonin induces the phosphorylation of receptor-interacting serine/threonine-protein kinase 1 (RIPK1)/RIPK3 and mixed lineage kinase domain-like kinase (MLKL). Treatment with RIPK1 inhibitors (necrostatin-1 or 7-Cl-O-Nec-1) significantly reversed acetylshikonin-induced MLKL phosphorylation and NSCLC cell death. These results indicate that acetylshikonin activated the RIPK1/RIPK3/MLKL cascade, leading to necroptosis in NSCLC cells. Our findings indicate that acetylshikonin reduces lung cancer cells by promoting G2/M phase arrest and necroptosis.

## INTRODUCTION

Lung cancer is a highly lethal disease with a poor prognosis [1]. Conventional treatments for lung cancer include surgery, chemotherapy, targeted therapy, and radiation therapy [2, 3]. Even though multiple tyrosine kinase inhibitors and immune checkpoint inhibitors have improved the situation of patients with NSCLC, these medicines still have drawbacks that make them effective for patients without specific target protein expression therapeutic potential is limited [3–5]. It is well known that the occurrence of tumors is related to the dysregulation of apoptosis or anti-apoptotic properties of cells. Many studies are devoted to the development of alternative mechanisms to promote cell death as targets for anticancer drug development [6]. Current studies have found that various forms of non-apoptotic programmed cell death (PCD), including necroptosis, can regulate cancer cell proliferation and tumor metastasis. This process is crucial to the efficacy of cancer therapy [7].

Necroptosis is a type of programmed cell death (PCD) that is activated by tumor necrosis factor, endoplasmic reticulum stress, DNA damage, and anticancer drugs. Necroptosis is initiated by the inhibition or inactivation of caspases [8–12]. Reactive oxygen species (ROS)-induced lipid peroxide production has also been shown to contribute to organelle membrane permeabilization and necroptosis signaling pathways [13]. Necroptosis activation is associated with a complex composed of receptor-interacting serine/threonine protein kinase 1 (RIPK1) and RIPK3, which results in the phosphorylation of mixed lineage kinase domain-like kinase (MLKL) [14]. The activation of MLKL increases the permeability of the cell membrane [15]. Several natural extracts, including β-lapachone and staurosporine, have been shown to induce necroptosis in cancer cells [16–19]. Shikonin has also been shown to induce ROS production, which leads to necroptosis in myeloid leukemia cells, lymphoma cells, and breast cancer cells [18, 20, 21].

Zicao (*Lithospermum erythrorhizon*) is a traditional Chinese medicine that has been used for centuries to treat local wounds [22]. Acetylshikonin is a naphthoquinone compound extracted from Zicao, which exhibits antioxidant, anti-inflammatory, and anticancer effects [23–31]. Research has shown that acetylshikonin induces apoptosis in hepatocellular carcinoma and oral squamous cell carcinoma by triggering the production of intracellular reactive oxygen species [32, 33]. Acetylshikonin treatment has also been shown to promote cell cycle arrest in the G2/M and S phases in chondrosarcoma and leukemia cells [34, 35]. Functional kinetics analysis suggests that the blockading of cellular drug transporters can be attributed to acetylshikonin increasing the sensitivity of multidrug-resistant human gastric and breast cancer cells to chemotherapy drugs [36]. Acetylshikonin also inhibits cell migration and invasion by reversing the epithelial-mesenchymal transition in triple-negative breast cancer cells [37]. Interestingly, in murine microglial cells and human neuroblastoma, acetylshikonin has been shown to protect against cell damage, induce the expression of antioxidant proteins, and suppress apoptosis [38, 39]. These results suggest that acetylshikonin exhibits a variety of pharmacological properties depending on the type of cell. This study explored the mechanisms underlying the effects of acetylshikonin on lung cancer cells.

In the current study, we examined the feasibility of using acetylshikonin for the treatment of lung cancer and explored the mechanisms underlying the observed effects. Acetylshikonin treatment was shown to significantly decrease the survival of lung cancer cells and increase membrane permeability. Treating lung cancer cells with acetylshikonin was also shown to promote cell death and cell cycle arrest by increasing intracellular ROS levels. Acetylshikonin-induced ROS production was associated with lipid peroxidation and inhibited glutathione peroxidase 4 (GPX4) expression. Increased phosphorylation of RIPK1/RIPK3 and MLKL activity indicates that acetylshikonin promoted necroptosis in lung cancer cells. Taken together, these results suggest the possibility of developing novel small-molecule drugs leveraging the effects of acetylshikonin on lung cancer cells.

## RESULTS

### Acetylshikonin suppressed cell growth and promoted cell death in lung cancer cells

**Figure 1:**
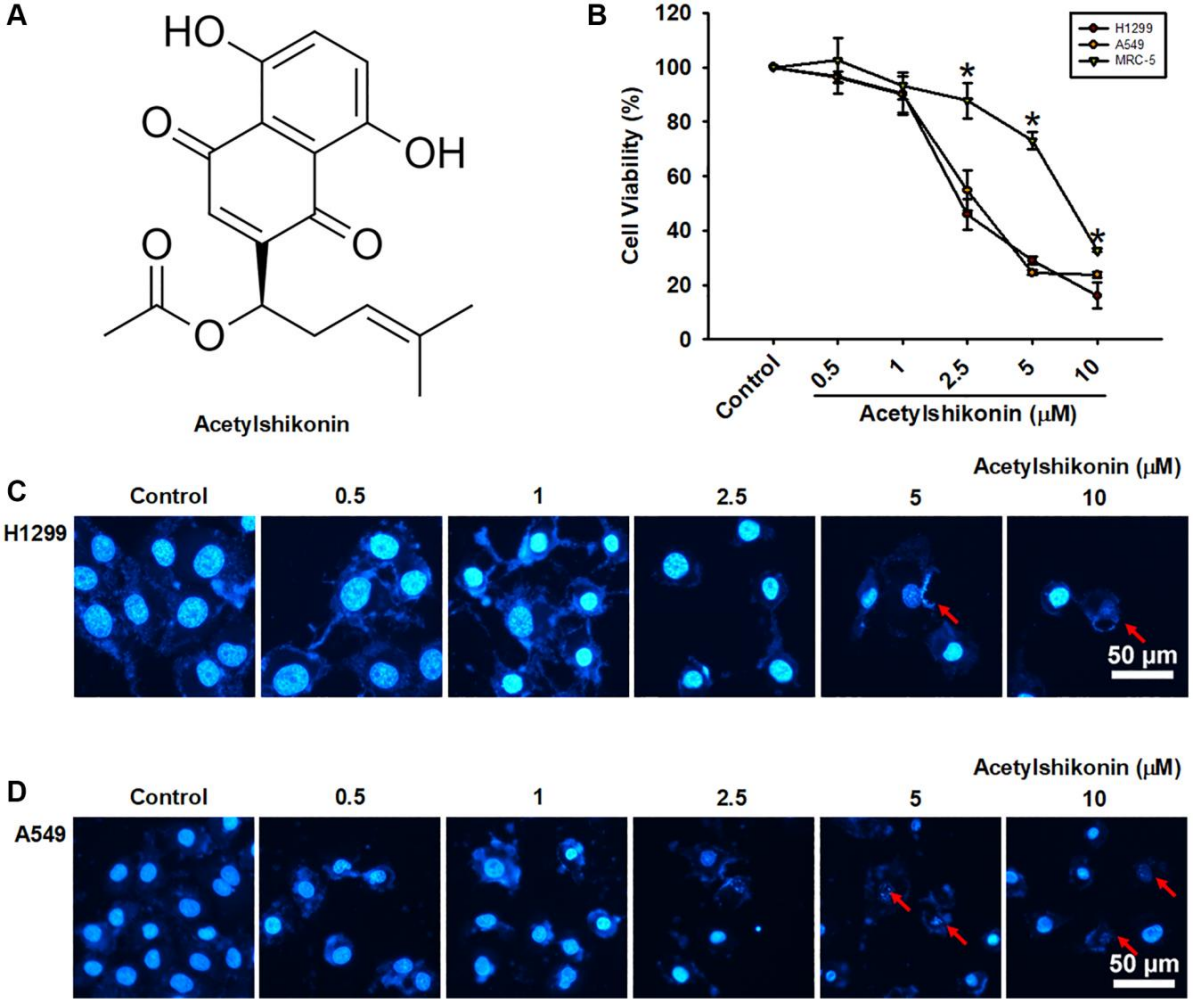
**Acetylshikonin decreased cancer cell viability and induced chromatin condensation and nuclear debris formation.** (**A**) Chemical structure of acetylshikonin. (**B**) CCK-8 assay results of MRC-5 cells and H1299 and A549 cells following incubation with acetylshikonin (0.5–10 μM) for 24 h (*n* = 4). (**C**, **D**) Fluorescence microscope images showing DAPI staining results and cell morphology of H1299 and A549 cells following treatment with acetylshikonin (0.5–10 μM). Red arrows indicated nuclear debris. Scale bar = 50 μm. MRC-5 cells and untreated cells were used as controls. Results are shown as means ± SD. ^*^p < 0.05 compared to untreated control.

We first analyzed the effects of acetylshikonin ((R)-1-(5,8-Dihydroxy-1,4-dioxo-1,4-dihydronaphthalen-2-yl)-4-methylpent-3-en-1-yl acetate; Figure 1A) on the viability of normal lung fibroblast cells (MRC-5) and lung cancer cells (H1299 and A549) using a CCK-8 assay. Acetylshikonin treatment resulted in IC50 values of 2.34 μM and 3.26 μM in H1299 and A549 cells, respectively (Figure 1B). This suggests that acetylshikonin could conceivably be used to treat cancer without causing significant damage to normal cells when administered in appropriate doses. Unlike classical apoptosis, DAPI staining results revealed that acetylshikonin treatment simultaneously led to chromatin condensation, the shrinkage of lung cancer cells, and an increase in observed cell debris (Figure 1C, 1D). These results indicate that acetylshikonin causes NSCLC cell death.

### Acetylshikonin promoted DNA fragmentation and cell cycle arrest in lung cancer cells in the G2/M phase

**Figure 2:**
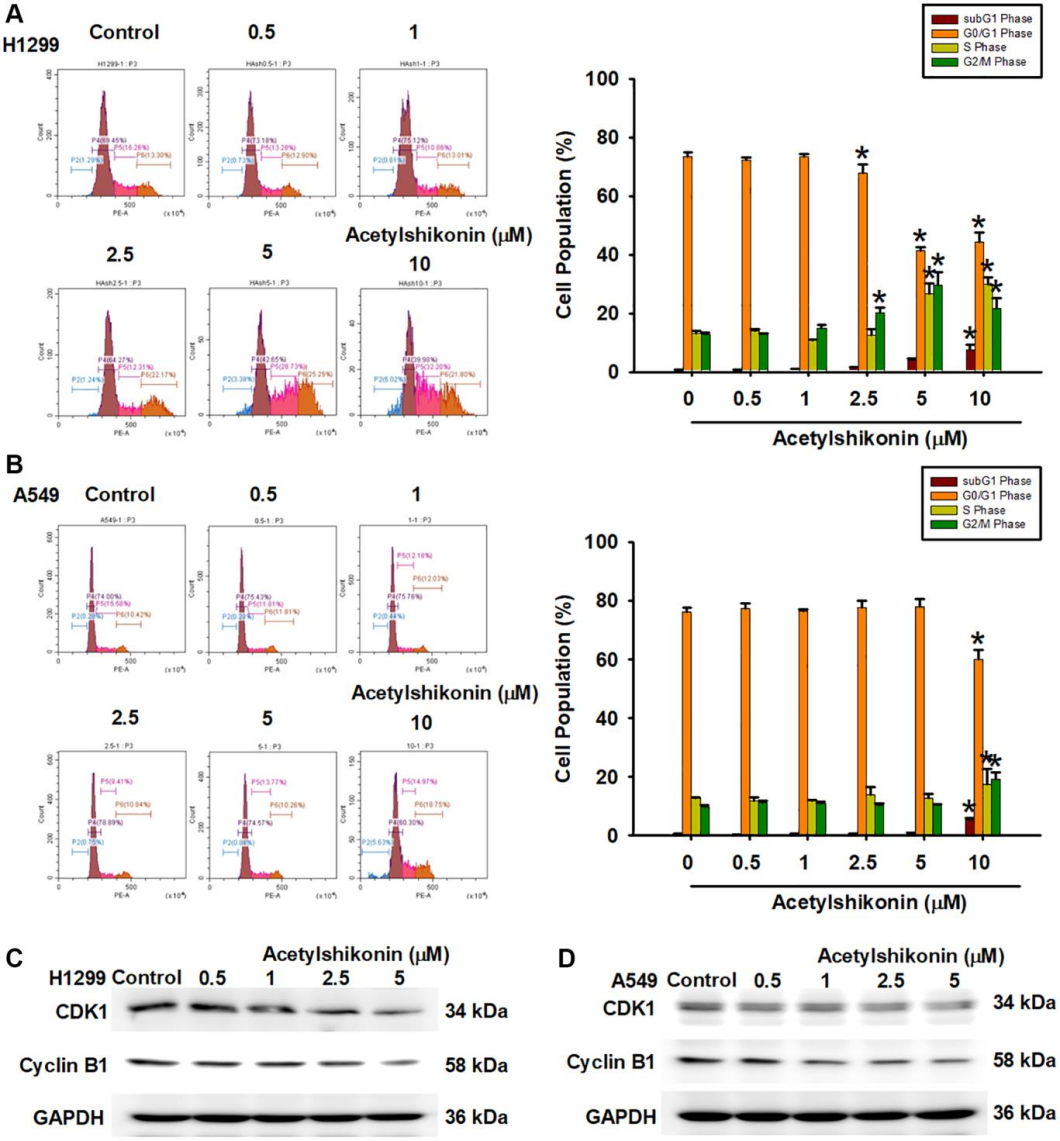
**Acetylshikonin promoted DNA fragmentation and cell cycle arrest in G2/M phase.** (**A**, **B**) Flow cytometry image results indicating cell cycle progression in H1299 and A549 cells following treatment with acetylshikonin (0.5–10 μM) for 24 h and PI staining for 30 min (*n* = 4). (**C**, **D**) Western blot analysis showing CDK1 and cyclin B1 protein expression in H1299 and A549 cells treated with acetylshikonin (0.5–10 μM) for 6 h (*n* = 4). Untreated cells were used as controls. Results are shown as means ± SD. ^*^p < 0.05 compared to untreated control.

We then examined the mechanisms underlying the inhibition of NSCLC cell proliferation by acetylshikonin. We hypothesized that acetylshikonin could induce cell cycle arrest and apoptosis. First, we analyzed the cell cycle in the acetylshikonin-treated A549 and H1299 cells using flow cytometry. Acetylshikonin was shown to significantly increase the proportion of NSCLC cells in the subG1 and G2/M phase, indicating that DNA strands were broken in NSCLC cells with cell cycle progression was arrested in G2/M phase (Figure 2A, 2B). Furthermore, we analyzed the cell cycle checkpoint proteins CDK1 and cyclin B1 (cell cycle regulator proteins) in the acetylshikonin-treated A549 and H1299 cells using western blotting. We found that acetylshikonin inhibited the expression of CDK1 and cyclin B1, which was consistent with our flow cytometry results (Figure 2C, 2D). These results highlight the role of acetylshikonin by promoting cell death and cell cycle arrest to inhibit cancer cell proliferation in the lung cancer therapy.

### Acetylshikonin promoted cell death by increasing membrane permeability resulting in NSCLC cells with swollen morphology

**Figure 3:**
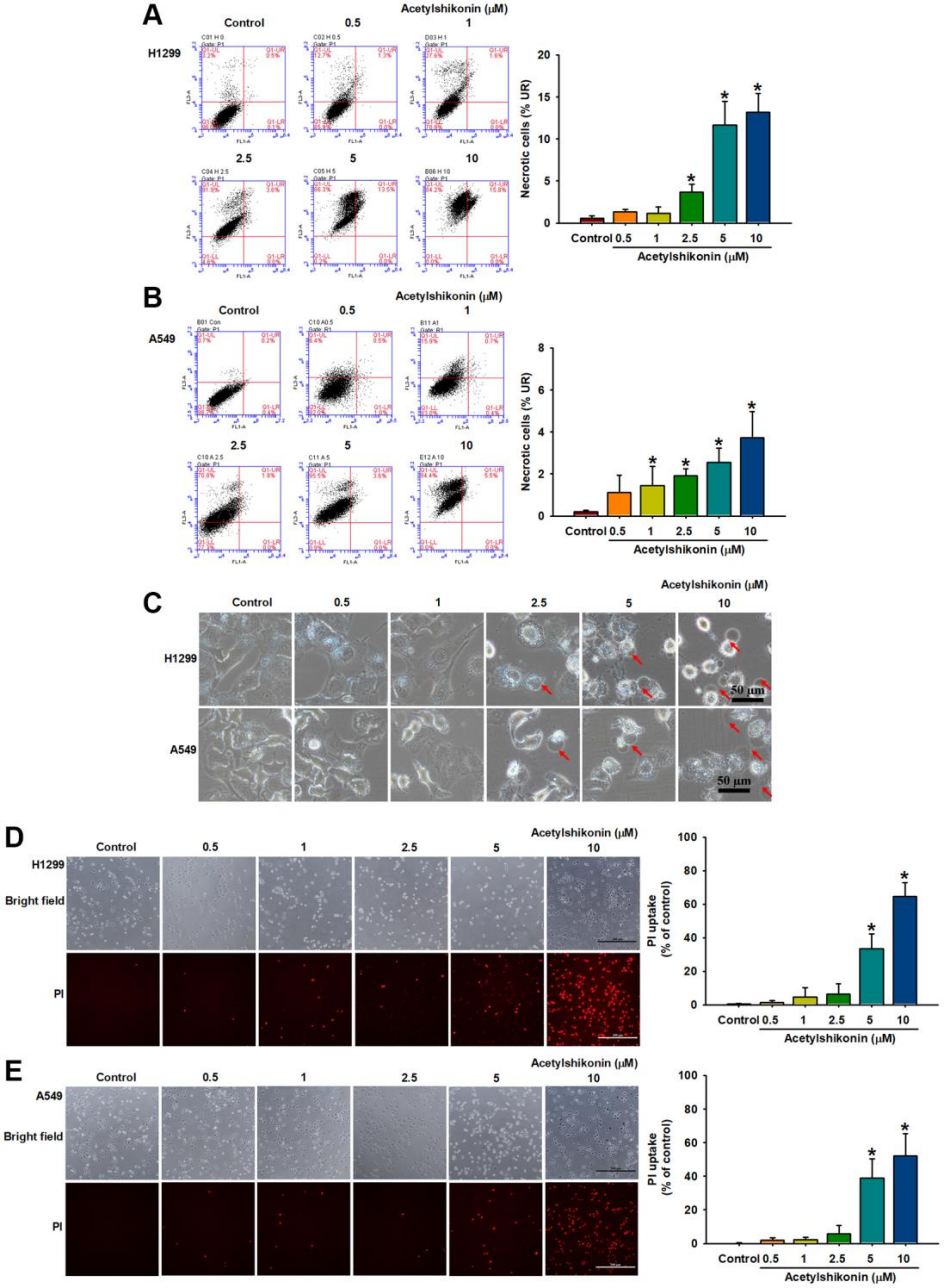
**Acetylshikonin increased the membrane permeability of NSCLC cells and the proportion of necrotic NSCLC cells.** (**A**, **B**) Flow cytometry results for Annexin V/PI showing the incidence of cell death among H1299 and A549 cells following treatment with acetylshikonin (0.5–10 μM) for 24 h (*n* = 4). (**C**) Phase microscope images of NSCLC cells following incubation with acetylshikonin (0.5–10 μM) for 24 h. Red arrows indicate swollen blebs. Scale bar = 50 μm. (**D**, **E**) PI staining results of H1299 and A549 cells following treatment with acetylshikonin (0.5–10 μM) for 4 h. Scale bar = 200 μm. Untreated cells were used as controls. Results are shown as means ± SD. ^*^p < 0.05 compared to untreated control.

The mechanism underlying acetylshikonin-induced lung cancer cell death was examined by performing Annexin V/PI staining followed by flow cytometry. Our results revealed that acetylshikonin increased the population of cells positive for Annexin V and PI in lung cancer cells (Figure 3A, 3B). In addition to necrosis, the increase in cell population positive for PI may be related to the increase in cell membrane permeability. This result prompted us to further observe the effect of acetylshikonin on the morphology and membrane permeability of cells. After treating lung cancer cells with acetylshikonin at indicated concentrations (0–10 μM) for 24 h, we observed swelling and bleb formation in NSCLC cells, with the number of blebs increasing in a dose-dependent manner (Figure 3C). PI staining was then used to observe the membrane permeability in cells. The intracellular fluorescence signal of PI gradually accumulated throughout the 4 h incubation period (Figure 3D, 3E), indicating a dose-dependent increase in the membrane permeability of NSCLC cells. These results suggest that the mechanism by which acetylshikonin causes lung cancer cell death may be related to altered membrane permeability.

### Acetylshikonin caused oxidative stress and mitochondrial depolarization in lung cancer cells

**Figure 4:**
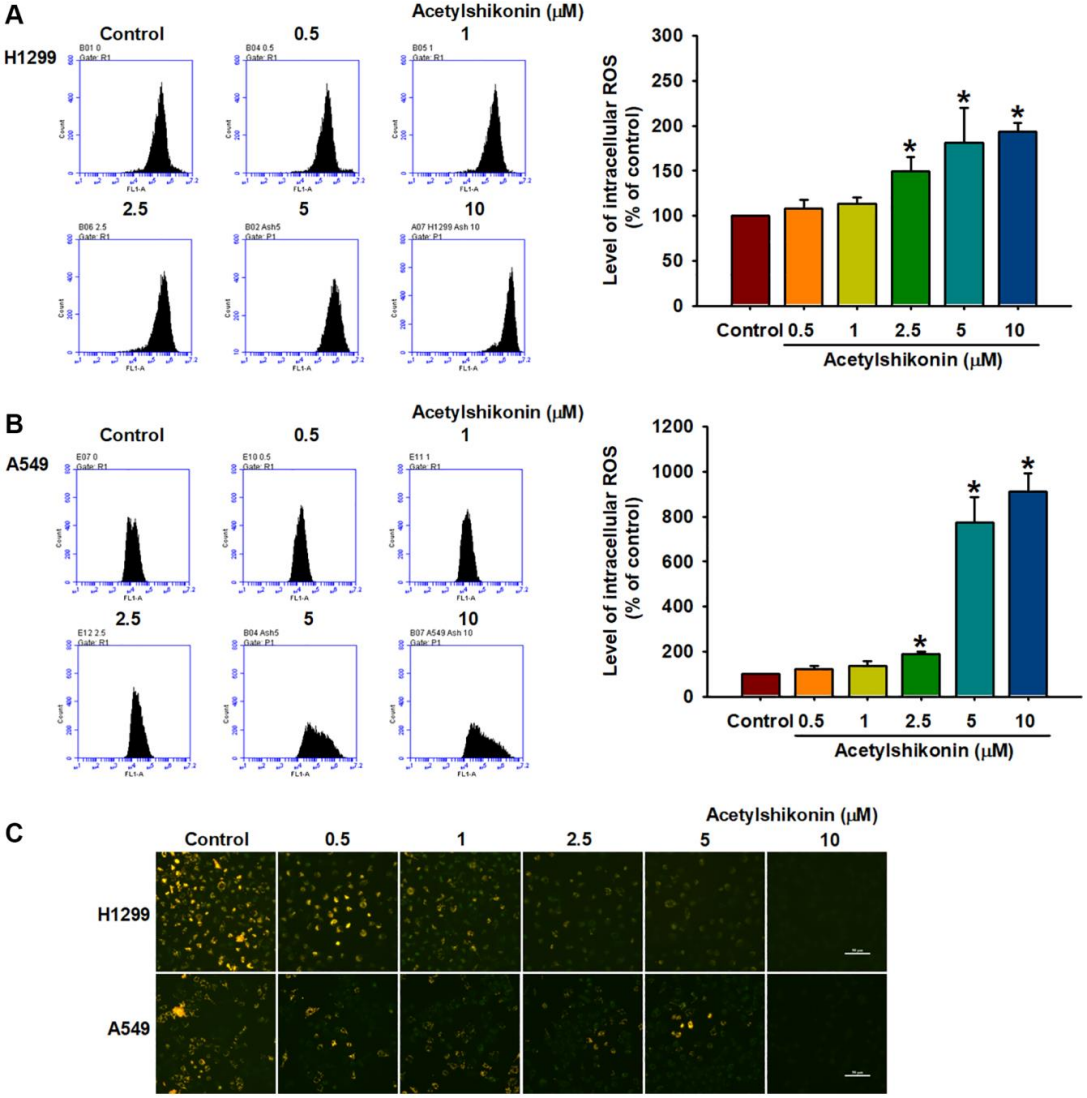
**Acetylshikonin induced intracellular ROS production and depolarization of mitochondrial membrane potential.** (**A**, **B**) Flow cytometry results indicating ROS production in H1299 and A549 cells following incubation with acetylshikonin (0.5–10 μM) and H_2_DCFDA for 30 min (*n* = 4). (**C**) Fluorescence microscope images used to analyze the MMP of NSCLC cells following incubation with acetylshikonin (0.5–10 μM) for 24 h and JC-1 staining for 30 min (*n* = 4). Scale bar = 50 μm. Untreated cells were used as controls. Results are shown as means ± SD. ^*^p < 0.05 compared to untreated control.

ROS is involved in the initiation of various types of PCD, including apoptosis and necroptosis [40]. In the current study, we investigated the effects of acetylshikonin on ROS production by measuring ROS levels using H_2_DCFDA in lung cancer cells. Our results revealed that acetylshikonin significantly increased ROS levels in lung cancer cells (Figure 4A, 4B). Furthermore, mitochondrial dysfunction is associated with altered intracellular ROS homeostasis and reduced MMP [41]. Our results using JC-1 staining to examine MMP revealed a reduction in aggregate JC-1 levels, indicating that acetylshikonin induced the depolarization of the mitochondrial membrane (Figure 4C). Taken together, these data suggest that acetylshikonin induces the death of lung cancer cells by increasing intracellular ROS levels and impairing mitochondrial function.

### Acetylshikonin caused lipid peroxidation and inhibited GPX4 expression in lung cancer cells

**Figure 5:**
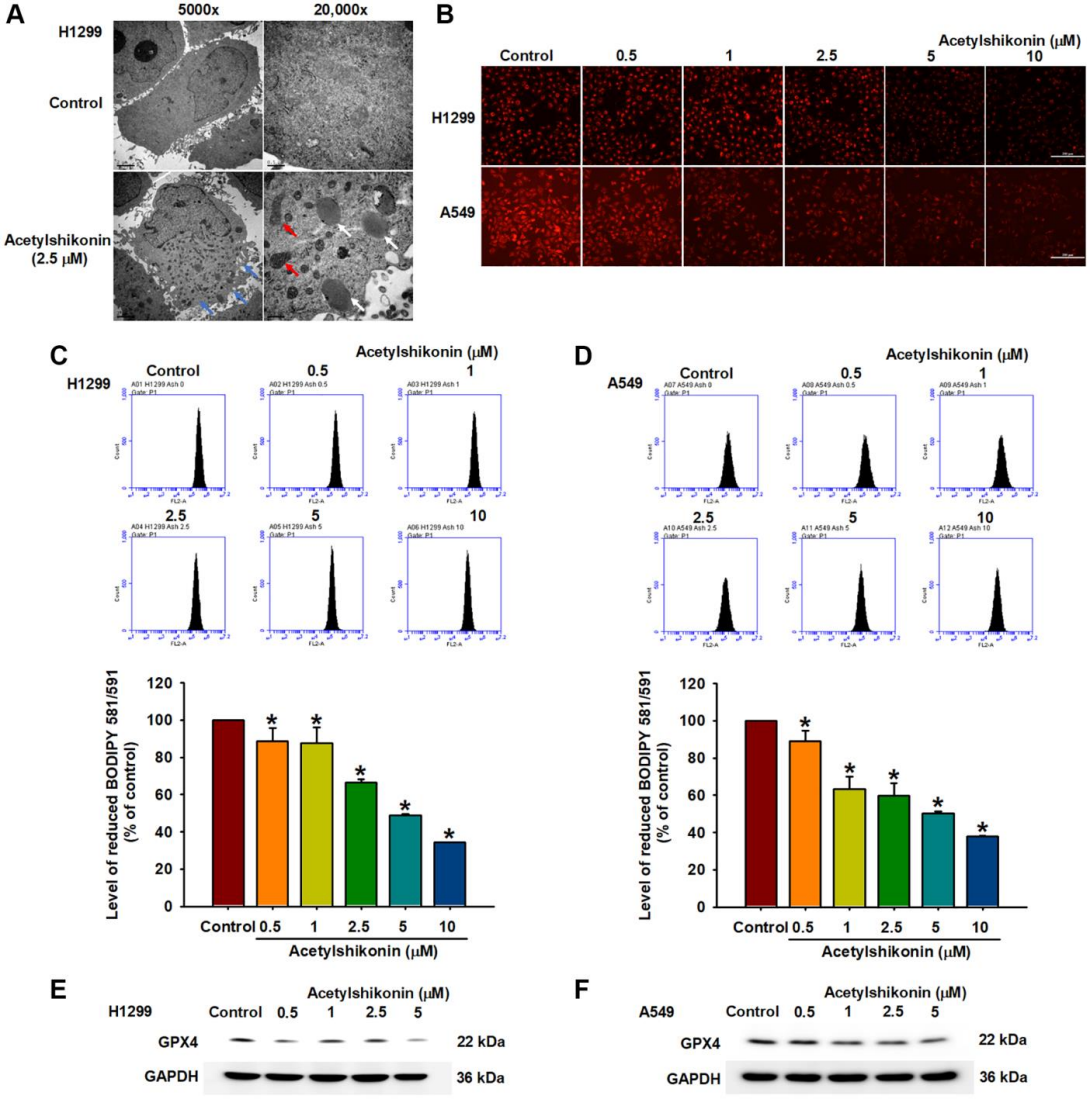
**Acetylshikonin induced necroptotic lipid peroxidation in NSCLC cells.** (**A**) Transmission electron microscopy analysis showing impaired membrane integrity (blue arrow) after treating H1299 cells with acetylshikonin for 6 h. The red arrow indicates mitochondria, and the white arrow indicates lysosomes. 5,000×: Scale bar = 2 μm. 20,000×: Scale bar = 0.5 μm. (**B**–**D**) Fluorescence microscope images and flow cytometry results indicating lipid peroxidation in H1299 and A549 cells incubated with acetylshikonin (0.5–10 μM) and BODIPY™ 581/591 C11 for 30 min (*n* = 4). Scale bar = 200 μm. (**E**, **F**) Western blot analysis showing GPX4 protein expression in H1299 and A549 cells following treatment with acetylshikonin (0.5–10 μM) for 24 h (*n* = 4). Untreated cells were used as controls. Results are shown as means ± SD. ^*^p < 0.05 compared to untreated control.

Cells use GPX enzymes to maintain homeostasis, resist lipid peroxidation, and prevent damage caused by oxidative stress [42]. Previous studies have reported that ROS-induced lipid peroxidation and the inhibition of GPX enzymes lead to necroptosis in colon cancer cells and neutrophils [43, 44]. In the current study, we sought to confirm whether acetylshikonin promotes ROS-induced intracellular lipid oxidation or inhibits GPX4 expression by analyzing cell morphology and protein expression. Transmission electron microscopy results revealed that acetylshikonin treatment caused cells to rupture, increased the number of lysosomes, and altered mitochondrial morphology (Figure 5A). C11-BODIPY staining revealed that acetylshikonin treatment led to the quenching of red fluorescence, indicating the oxidation of lipids in lung cancer cells (Figure 5B–5D). Consistent with these findings, we observed a decrease in GPX4 expression in NSCLC cells following treatment with acetylshikonin (Figure 5E, 5F). Taken together, these results suggest that acetylshikonin-induced lung cancer cell death is related to lipid peroxidation.

### Acetylshikonin-induced necroptosis via RIP kinases and MLKL in lung cancer cells

**Figure 6:**
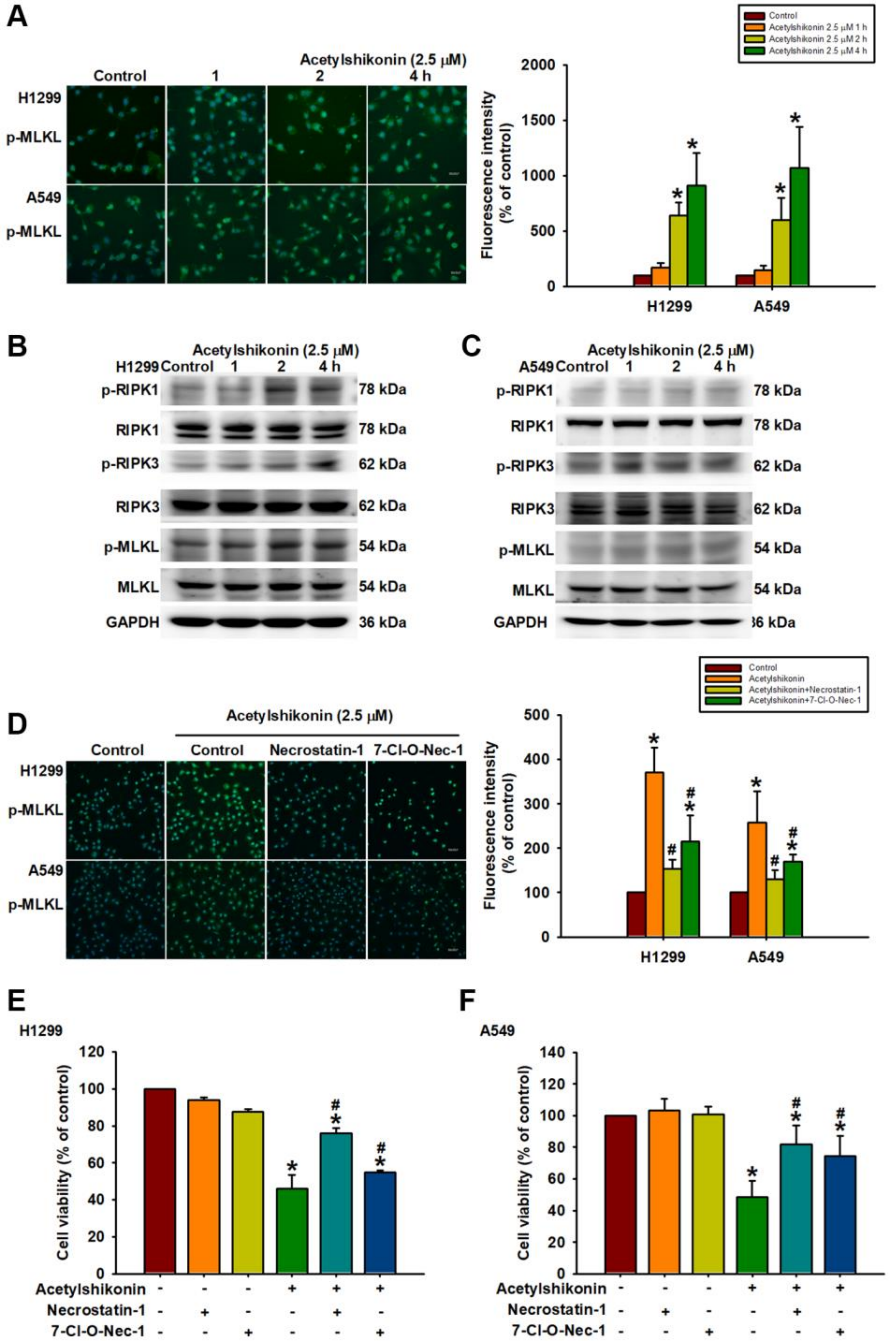
**Acetylshikonin promoted cell death via necroptotic RIPK1, RIPK3, and MLKL signaling activation.** (**A**) Fluorescence microscope images showing MLKL phosphorylation in H1299 and A549 cells following incubation with acetylshikonin (2.5 μM) for 0–4 h. Scale bar = 50 nm. (**B**, **C**) Western blot analysis showing RIPK1, RIPK3, and MLKL protein phosphorylation levels in NSCLC cells treated with acetylshikonin (2.5 μM) for 0–4 h (*n* = 4). (**D**) Fluorescence microscope images showing MLKL phosphorylation in H1299 and A549 cells preincubated with necrostatin-1 (20 nM) and 7-Cl-O-Nec-1 (30 nM) for 1 h and then incubated with acetylshikonin (2.5 μM) for a further 4 h. Scale bar = 50 nm. (**E**, **F**) CCK-8 assays indicating the viability of H1299 and A549 cells preincubated with necrostatin-1 (20 nM) and 7-Cl-O-Nec-1 (30 nM) for 1 h and then incubated with acetylshikonin (2.5 μM) for a further 24 h (*n* = 4). Untreated cells were used as controls. Results are shown as means ± SD. ^*^p < 0.05 compared to untreated control. ^#^*p* < 0.05 compared to acetylshikonin alone group.

Necroptosis is mediated by the RIPK1 and RIPK3 complex, which promotes MLKL phosphorylation, leading to increased membrane permeability and cell swelling resulting in cell death [45]. In the current study, we sought to determine whether the mechanism by which acetylshikonin induces lung cancer cell death is related to necroptosis by observing the phosphorylation of MLKL via immunofluorescence staining at various time intervals after treating cells with 2.5 μM acetylshikonin. Our results revealed that acetylshikonin increased MLKL phosphorylation (Figure 6A), which was further confirmed by Western blot analysis. We also determined that acetylshikonin activated RIPK1, RIPK3, and MLKL in NSCLC cells (Figure 6B, 6C). Furthermore, pretreatment with RIPK1 inhibitors (necrostatin-1, 20 nM, and 7-Cl-O-Nec-1, 30 nM) attenuated acetylshikonin-induced MLKL phosphorylation (Figure 6D). These results prompted the use of RIPK1 inhibitors to confirm whether acetylshikonin caused NSCLC cell death by inducing necroptosis. Indeed, pretreatment with RIPK1 inhibitors significantly reversed the viability of acetylshikonin-suppressed NSCLC cells (Figure 6E, 6F). Taken together, these results suggest that acetylshikonin activates the necroptosis pathway via the RIPK1/RIPK3/MLKL axis in lung cancer cells.

## DISCUSSION

Apoptosis is a cell death program that regulates cell numbers during normal physiology and disease. Defects in apoptosis are the basis of tumorigenesis and are more related to the failure of chemotherapy [46]. Therefore, resistance to cell death is one of the hallmarks of tumor cells [47]. In addition to conventional chemotherapy and targeted anticancer agents, researchers are trying to find small molecule drugs to induce cancer cell death through alternative apoptotic pathways as a novel therapeutic mechanism [7, 48].

The extraction of bioactive compounds from natural products has been the conventional approach for treating diseases, including cancer [49]. Nonetheless, despite the development of numerous antitumor drugs, cancer remains a leading cause of death in humans [50]. The absence of adverse side effects in numerous traditional Chinese medicines has made them a major focus of anticancer research [51]. This study highlighted the anticancer effects of acetylshikonin in suppressing cell proliferation, promoting cell cycle arrest, increasing ROS levels leading to an imbalance of mitochondrial membrane potential, and inducing necroptosis in NSCLC cells.

Acetylshikonin has been shown to induce apoptosis in oral cancer cells, leukemia cells, and colorectal cancer cells [33, 35, 52]. In the current study, DAPI staining revealed acetylshikonin-induced morphological changes in NSCLC cells, including shrinkage, chromatin condensation, and the formation of debris. Annexin V/PI staining also revealed an increase in the number of PI-positive and partial Annexin V-positive cell populations, leading us to speculate that acetylshikonin-induced cancer cell death may be associated with other forms of PCD. Phase-contrast microscopy revealed that after exposure to acetylshikonin, NSCLC cells underwent swelling and bleb formation. As shown in Figure 2D, PI staining of cells revealed the dose-dependent accumulation of PI-positive NSCLC cells. The fact that PI cannot enter healthy live cells implies that acetylshikonin compromised the integrity of the cell membrane to allow PI uptake. These findings are consistent with previous reports of increased swelling and permeability characteristics on necroptosis [53]. These results that prompted our investigation of whether the anticancer effects of acetylshikonin were related to necroptosis.

We also investigated the effects of acetylshikonin on the viability and proliferation of NSCLC cells. Following treatment with acetylshikonin, the viability of NSCLC cells was significantly lower than that of normal lung cells. Acetylshikonin was also shown to cause cell cycle arrest in the G2/M phase. Note that this was confirmed by assessing the expression of cell cycle regulatory proteins via western blot analysis. Interestingly, these findings are consistent with those in previous studies on chondrosarcoma cells [34], but not in studies on colorectal cancer, in which cell cycle arrest occurred in the G1 phase [52]. These inconsistencies pertaining to the anticancer effects of acetylshikonin deserve further consideration in the context of lung cancer.

Excessive ROS production has been shown to cause PCD, including necroptosis [13, 54, 55]. ROS levels have been implicated in various forms of necroptosis is characterized by a decrease in the expression and activity of GPX enzymes and lipid peroxidation [56]. Studies on leukemia cells and oral squamous cell carcinoma have reported that acetylshikonin treatment can lead to elevated ROS levels resulting in cell death [33, 35]. We observed an acetylshikonin-induced increase in ROS levels in NSCLC cells. BODIPY™ 581/591 C11 staining also revealed that acetylshikonin induced lipid peroxidation, as indicated by a shift in the excitation and emission spectra from 581/591 nm to 488/510 nm. Western blot analysis revealed that acetylshikonin negatively regulated the expression of GPX4 enzyme. Taken together, we hypothesize that acetylshikonin initiates necroptosis by inducing oxidative stress in NSCLC cells.

Apoptotic evasion of cancer cells is a major challenge in cancer treatment [57], at least partially attributable to the treatment resistance and recurrence. This has prompted alternative approaches to induce cancer cell death, i.e., not involving apoptotic pathways [58]. In the current study on NSCLC cells, acetylshikonin treatment led to RIPK1/RIPK3/MLKL phosphorylation, which is a crucial step in triggering the necroptosis signaling pathway [53, 59]. We also determined that acetylshikonin promoted the distribution of activated MLKL to the cytoplasm and was associated with increased membrane permeability [15]. These findings suggest that acetylshikonin induces necroptosis in NSCLC cells by activating the RIPK1/RIPK3/MLKL signaling pathway. In the current study, immunofluorescence staining revealed that acetylshikonin-induced MLKL phosphorylation was suppressed in cells pretreated with necrostatin-1 and 7-Cl-O-Nec-1. These inhibitors also significantly attenuated the acetylshikonin-induced decrease in cell viability. These results provide further evidence that in NSCLC cells, the anticancer effects of acetylshikonin involve the induction of necroptosis.

This study demonstrated the anticancer effects of acetylshikonin in NSCLC cells. We determined that even low doses of acetylshikonin reduced the viability of lung cancer cells without significantly affecting normal cells. When used to treat lung cancer, acetylshikonin was shown to promote cell death and arrest cell cycle progression in the G2/M phase. Incubation with acetylshikonin was also shown to increase ROS levels, which led to MMP dysfunction and lipid peroxidation. Finally, acetylshikonin was found to increase membrane permeability and induce necroptosis by downregulating GPX4 expression and promoting the phosphorylation of RIPK1, RIPK3, and MLKL. Our findings suggest that acetylshikonin-based ability to induce necroptosis may facilitate the development of small molecule compounds for cancer therapy.

## MATERIALS AND METHODS

### Chemicals

Primary antibodies specific to the following proteins were purchased from Genetex (Irvine, CA, USA): receptor-interacting protein kinase 1 (RIPK1), phospho-RIPK1 (phospho Tyr384), RIPK3, phospho-RIPK3 (phospho Ser232), mixed lineage kinase domain-like pseudokinase (MLKL), phospho-MLKL (phospho Ser358), glutathione peroxidase 4 (GPX4), and glyceraldehyde-3-phosphate dehydrogenase (GAPDH). Cyclin-dependent kinase 1 (CDK1) and cyclin B1 were purchased from Merck Millipore (Burlington, MA, USA). Anti-rabbit polyclonal antibodies and anti-mouse monoclonal antibodies were purchased from Santa Cruz Biotechnology (Dallas, TX, USA). 7-Cl-O-Nec1 was purchased from Abcam (Cambridge, MA, USA). All other chemicals were purchased from Sigma-Aldrich (St. Louis, MO, USA).

### Cell culture

The human non-small cell lung cancer (NSCLC) cell line H1299 was obtained from the American Type Culture Collection (Manassas, VA, USA). The NSCLC cell line A549 and normal lung fibroblast cell line MRC-5 were obtained from the Bioresource Collection and Research Center (Hsinchu, Taiwan). All cells were cultured in accordance with suppliers’ recommendations. H1299 cells were cultured in Roswell Park Memorial Institute 1640 medium supplemented with 10% fetal bovine serum, 100 U/mL penicillin, and 100 μg/mL streptomycin. A549 cells were cultured in Ham’s F-12 nutrient mixture supplemented with the above-mentioned supplements, whereas MRC-5 cells were cultured in Eagle’s minimum essential medium with the same supplements. Cells were incubated in an incubator under 5% CO_2_ in air at 37°C.

### Cell viability assay

Cells were seeded at a density of 1 × 10^4^ cells per well in 48-well plates and allowed to attach overnight. The cells were then treated with the indicated concentrations of acetylshikonin for 24 h. Cell viability was evaluated using a cell counting kit-8 (CCK-8; Sigma-Aldrich, St. Louis, MO, USA) after incubating the cells at 37°C for 4 h. The optical density was measured using a spectrophotometer at 450 nm (BioTek, Winooski, VT, USA).

### Chromatin condensation analysis

Chromatin condensation was monitored using 4,6-diamidino-2-phenylindol (DAPI; Merck Millipore, Burlington, MA, USA). This involved treating cells with various doses of acetylshikonin (0.5, 1, 2.5, 5, and 10 μM) for 24 h. Fixed cells were then incubated with DAPI solution (1 μg/mL) for 5 min, after which the nuclear morphology was observed using an Eclipse Ti fluorescence microscope (Nikon, Tokyo, Japan).

### Analysis of apoptotic and necrotic cells

After treatment with acetylshikonin for 24 h, apoptotic and necrotic cells were identified by performing Annexin V/propidium iodide (PI) assays (Sigma-Aldrich, St. Louis, MO, USA). This involved harvesting and staining live cells in accordance with the manufacturer’s instructions using 1 μg/mL PI and 0.025 μg/mL FITC-conjugated Annexin V. Staining was performed in the dark at room temperature for 15 min, after which the cells were analyzed using a flow cytometer (Accuri C5, BD, East Rutherford, NJ, USA).

### Analysis of cell membrane permeability

Following adhesion, cells (5 × 10^4^) were treated with the indicated concentrations of acetylshikonin for 4 h, after which PI uptake analysis was performed to identify changes in the membrane permeability of cells. Alterations were examined using a Nikon Eclipse Ti fluorescence microscope (Nikon, Japan).

### Cellular ROS assay

Intracellular ROS production was examined by treating cells (5 × 10^5^) with acetylshikonin at various concentrations, followed by the addition of H_2_DCFDA (Thermo Fisher Scientific, Waltham, MA, USA) at a concentration of 1 μM. The mixture was then incubated at 37°C for 30 min, and intracellular ROS production was measured by using a flow cytometer (Accuri C5, BD, East Rutherford, NJ, USA).

### Analysis of mitochondrial membrane potential

The mitochondrial membrane potential (MMP) and polarity transition (both of which are related to cell damage and apoptosis) were assessed using JC-1 dye (Thermo Fisher, Waltham, MA, USA). Cells were treated with acetylshikonin for 24 h and then incubated with JC-1 (5 μg/mL) for 30 min. Fluorescence images were captured using a fluorescence microscope (Nikon, Japan).

### Cell cycle analysis

Cell cycle progression was observed by seeding cells (5 × 10^5^) in 6-well plates followed by treatment with acetylshikonin at various concentrations for 24 h. The cells were then harvested and stained using a PI solution (0.1% Triton X-100, RNase A 0.2 mg/ml, PI 10 μg/mL; Sigma-Aldrich, St. Louis, MO, USA) which were analyzed using a flow cytometer (Accuri C5, BD, East Rutherford, NJ, USA).

### Immunoblotting analysis

Proteins separated using sodium dodecyl sulfate-polyacrylamide gel electrophoresis were transferred to Immobilon polyvinylidene difluoride membranes (Merck Millipore, Burlington, MA, USA). The membranes were blocked using 5% non-fat milk in Tris-buffered saline with Tween 20 (TBST) and incubated with primary antibodies (diluted 1:1,000) overnight at 4°C. The blots were then washed using TBST and incubated with anti-rabbit peroxidase-conjugated secondary antibodies (diluted 1:10,000) at room temperature for 1 h. Protein signals were detected using enhanced chemiluminescence and visualized using a UVP chemiluminescence detection system (Analytik Jena US, Upland, CA, USA).

### Transmission electron microscopy analysis

After treatment with acetylshikonin for 6 h, H1299 cells (5 × 10^5^) were trypsinized after washing to remove residual medium. The suspended cells were immediately fixed in 70% Karnovsky fixative at 4°C until embedding and then observed under a JEOL JEM-1400 transmission electron microscope (Tokyo, Japan) to examine ultrastructural changes.

### Analysis of lipid peroxidation

Adhered cells (5 × 10^4^) were incubated with acetylshikonin and BODIPY™ 581/591 C11 (Thermo Fisher Scientific, Waltham, MA, USA) at 37°C for 30 min. In a reduced state, the excitation and emission wavelengths of BODIPY™ 581/591 dye were 581/591 nm. Following oxidation, the excitation and emission wavelengths of the dye shifted to 488/510 nm. After 30 min, the cell culture medium was removed, and the cells were washed twice using PBS. Lipid peroxidation was examined via fluorescence microscopy and flow cytometer.

### Immunofluorescence analysis

After treatment, cells were fixed and incubated with a specific primary antibody (1:200) specific for phospho-MLKL at 4°C overnight. After the primary antibody was removed, the cells were washed and incubated with a secondary goat anti-rabbit IgG antibody (DyLight488, Genetex, Irvine, CA, USA) for 1 h at room temperature. Cells were then incubated with a DAPI solution for 5 min and examined using a fluorescence microscope (Nikon, Japan).

### Statistical analysis

Results are expressed as mean ± standard deviation (SD). Statistical analysis for multiple groups was conducted using one-way ANOVA followed by the Fisher-LSD post-hoc test. A *p*-value of less than 0.05 was considered statistically significant.

### Data availability

The datasets generated for this study can be accessed upon request to the corresponding author.
